# Tongue Thickness Relates to Nutritional Status in the Elderly

**DOI:** 10.1007/s00455-012-9407-z

**Published:** 2012-04-27

**Authors:** Fumiyo Tamura, Takeshi Kikutani, Takashi Tohara, Mitsuyoshi Yoshida, Ken Yaegaki

**Affiliations:** 1 Rehabilitation Clinic for Speech and Swallowing Disorders, The Nippon Dental University School of Life Dentistry at Tokyo, Dental Hospital, 3-16, Fujimi 2-chome, Chiyoda-ku, Tokyo, 102-8158 Japan; 2 Division of Clinical Oral Rehabilitation, The Nippon Dental University Graduate School of Life Dentistry at Tokyo, 9–16, Fujimi 1-chome, Chiyoda-ku, Tokyo 102-0071, Japan; 3 Dental Department, Hiroshima City General Rehabilitation Center, Hiroshima, Japan; 4 Department of Hygiene, The Nippon Dental University School of Life Dentistry at Tokyo, Tokyo, Japan

**Keywords:** Tongue thickness, Nutritional status, Dysphagia, Sarcopenia, Ultrasonography, Deglutition, Deglutition disorders

## Abstract

Many elderly people under long-term care suffer from malnutrition caused by dysphagia, frequently leading to sarcopenia. Our hypothesis is that sarcopenia may compromise oral function, resulting in dysphagia. The objectives of this study were to evaluate sarcopenia of the lingual muscles by measuring the tongue thickness, and elucidate its relationship with nutritional status. We examined 104 elderly subjects (mean age = 80.3 ± 7.9 years). Anthropometric data, such as triceps skinfold thickness and midarm muscle area (AMA), were obtained. The tongue thickness of the central part was determined using ultrasonography. Measurement was performed twice and the mean value was obtained. The relationship between tongue thickness and nutritional status was analyzed by Pearson’s correlation coefficient and Spearman’s rank correlation coefficient. AMA and age were identified by multiple-regression analysis as factors influencing tongue thickness. The results of this study suggest that malnutrition may induce sarcopenia not only in the skeletal muscles but also in the tongue.

The tongue plays an important role in feeding and swallowing function. Feinberg et al. [1] reported that bolus misdirection due to dysfunction and abnormality was more frequent at the oral stage alone or at both the oral and pharyngeal stages than at the pharyngeal stage alone. Dysfunction and abnormality of the tongue might also be a reason for dysphagia, since problems at the oral stage are one of the reasons for dysphagia. Many elderly people under long-term care suffer from malnutrition caused by dysphagia and frequently develop sarcopenia because of malnutrition [2]. Sarcopenia is defined as loss of muscular mass, strength, and physical performance. Sarcopenia caused by aging is also affected by the levels of anabolic hormones, which may suppress appetite or lead to a reduction of protein synthesis, resulting in worsening of the condition [3, 4] and subsequent restriction of physical activities in the elderly.

Elderly people frequently suffer from eating malfunction and malnutrition [5, 6]. Fewer occluding pairs of teeth decrease chewing function and increase chewing difficulty [7]. Therefore, chewing ability may contribute to the regulation of nutritional status in the elderly, as reported previously [8]. Subsequently, chewing ability is associated with not only oral health status but also with the physical constitution of the elderly [8]. Low tongue pressure reflects dysphagic tongue movement and cough [9]. Moreover, a decline of oral muscle strength as well as fewer occluding teeth may cause malfunction of feeding; therefore, we presume that malnutrition may worsen in dysphagic patients. Our hypothesis is that sarcopenia may occur in the tongue as well as in other tissues. In other words, we speculated that muscle volume may relate to tongue sarcopenia rather than to body size. If so, sarcopenia of the lingual muscles would compromise oral function in the elderly. Once atrophy of the tongue occurs, people may start to develop malnutrition because of dysphagia. In most cases, the meal texture of these people becomes softer, requiring less power of tongue movement. Consequently, tongue atrophy may be promoted. The objectives of this study were to evaluate sarcopenia of the lingual muscles by measuring the tongue thickness and to elucidate its relationship with nutritional status.

## Subjects and Methods

We studied 104 elderly subjects (32 men and 72 women, mean age = 80.3 ± 7.9 years). All maintained occlusal support with either natural dentition or dentures. Neither paralysis nor atrophy of the tongue was observed. The anthropometric data of triceps skinfold thickness (TSF), midarm muscle area (AMA), body weight (BW), and height (HT) were measured to evaluate nutritional status [8, 10].

**Fig. 1:**
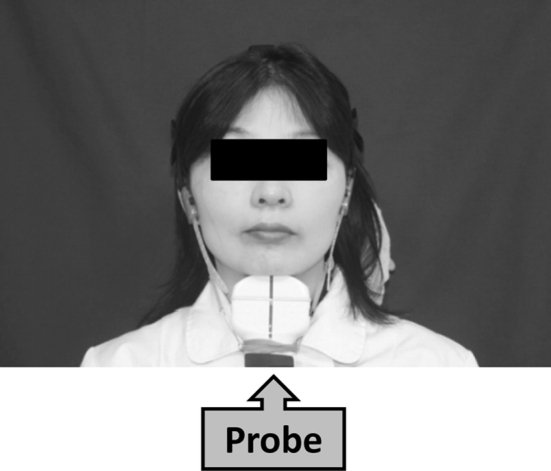
Position of ultrasonic probe in frontal view

Anthropometric measurements were conducted as follows: Mid-upper-arm circumference (MAC) was measured on the left arm with a tape measure. TSF was measured with Harpenden Skinfold Calipers over the triceps muscle at the midway point between the acromion and the olecranon process. AMA was calculated from MAC and TSF values based on a previously reported formula [11]. The mean of the twice-repeated measurements was taken as the true value. Tongue thickness was measured using ultrasonography (Nemio 17, SSA-550A, Toshiba Medical Systems, Tokyo, Japan). A fixation device to retain a 3.75-MHz convex probe (contact face size = 12 × 70 mm) in an appropriate position was employed to obtain accurate images, as shown in Fig. 1. To assure stable image acquisition, the probe was firmly fixed to the subject’s lower jaw by wrapping a belt around the head. The subjects were asked to remain seated in an upright position. They were also instructed to swallow their saliva often and to set the tongue at the resting position. Then, ultrasonic measurements were carried out.

**Fig. 2:**
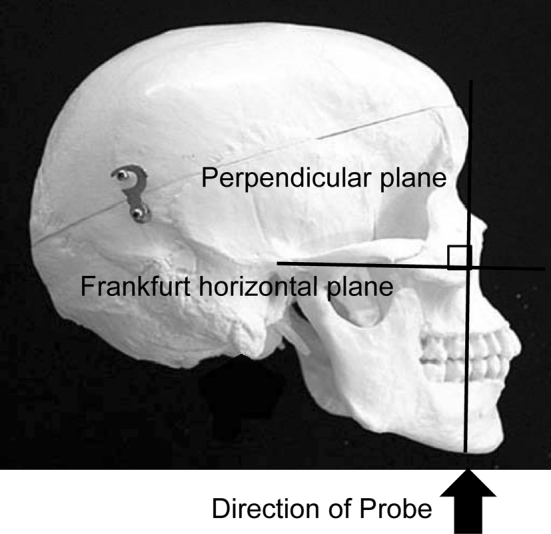
Position of ultrasonic probe in lateral view. The measurement points were determined at the center of the plane perpendicular to the Frankfurt horizontal plane in a frontal section. The perpendicular plane passes through the distal surfaces of the mandibular second premolars on both sides

**Fig. 3:**
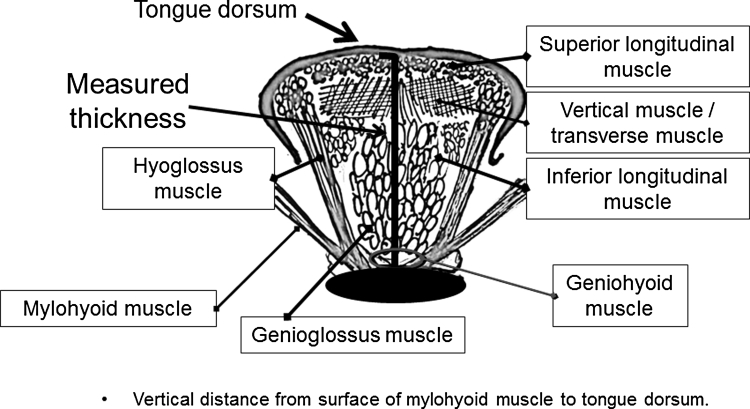
Diagram of tongue. Measured thickness is the vertical distance from the surface of mylohyoid muscle to the tongue dorsum

**Fig. 4:**
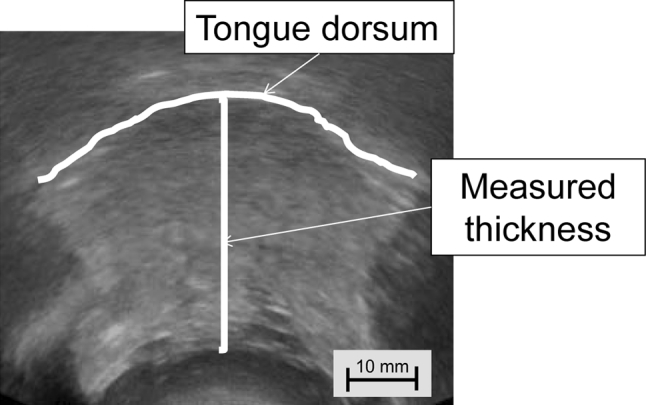
Ultrasonographic image

The measurement points were determined on the upper and lower surfaces of the lingual muscles in the center of the plane perpendicular to the Frankfurt horizontal plane in a frontal section, as shown in Fig. 2 [12]. This perpendicular plane went through the distal surfaces of the mandibular second premolars on both sides. The measurement point on the coronal plane is shown in Fig. 3. The vertical distance was measured from the surface of the mylohyoid muscle to the tongue dorsum. Figure 4 shows an image of a frontal section of the tongue on ultrasonography. Measurements were performed twice in freeze-frame when the tongue was restored to the resting position after swallowing saliva, and the mean values were obtained. To determine the reliability of the tongue thickness measurement, the two-way mixed-effects model of the intraclass correlation coefficient (ICC) (1,2) was used. The ICC values were above 0.75, indicating good reliability; values of 0.9 and above are reportedly even more reliable for ensuring the validity and reproducibility of clinical measurements [13]. The ICC (1,2) value for the intrarater reliability of tongue thickness measurement was 0.856 (95 % CI: 0.741–0.924).

The relationship between tongue thickness and nutritional status was analyzed using Pearson’s correlation coefficient and Spearman’s rank correlation coefficients using the software SPSS v16 (SPSS, Inc., Chicago, IL).

This study was approved by the Ethics Committee of The Nippon Dental University, School of Life Dentistry at Tokyo, Dental Hospital. Before starting measurements, the purpose and the protocol were explained to the subjects and/or their guardians in order to obtain their consent.

## Results

### Baseline Characteristics of Subjects

Table 1 gives the baseline characteristics of our subjects. TSF = 11.4 ± 4.6 mm, AMA = 34.9 ± 7.6 cm^2^, HT = 151.2 ± 8.8 cm, BW = 48.9 ± 8.8 kg, and tongue thickness = 46.9 ± 5.5 mm.

**Table 1:** Baseline characteristics of subjects (*n* = 104)

	Mean	SD
Age (years)	80.3	7.9
TSF	11.4	4.6
AMA	34.9	7.6
Height (cm)	151.2	8.8
Body weight (kg)	48.9	8.8
Tongue thickness (mm)	46.9	5.5

*TSF* triceps skinfold thickness, *AMA* arm muscle area

### Correlation Coefficients Between Tongue Thickness and Other Variables

Table 2 gives the correlation coefficients between tongue thickness and the other variables examined. Tongue thickness correlated with age (*r* = − 0.393, *P* < 0.001), TSF (*r* = 0.225, *P* < 0.05), AMA (*r* = 0.424, *P* < 0.001), HT (*r* = 0.312, *P* < 0.01), and BW (*r* = 0.434, *P* < 0.001).

**Table 2:** Pearson’s rank correlation coefficient between tongue thickness and other variables

Variables	Coefficient	*P* value
Age	−0.393	0.000
TSF	0.225	0.022
AMA	0.424	0.000
Height	0.312	0.001
Body weight	0.434	0.000

*TSF* triceps skinfold thickness, *AMA* arm muscle area

### Stepwise Multiple Regression Analysis

Table 3 shows the results of a stepwise multiple regression analysis conducted to identify the factor most strongly influencing tongue thickness. The multiple correlation coefficient (*R*) was 0.492 and the adjusted coefficient of determination (*R* ^2^) was 0.227.

**Table 3:** Factors related to tongue thickness by stepwise multiple regression analysis

Variables	Beta	*t*	*P* value
AMA	0.231	3.412	0.001
Age	−0.188	−2.868	0.005

*AMA* arm muscle area

Model 1: Multiple correlation coefficient (*R*) = 0.424; adjusted coefficient of determination (*R* ^2^) = 0.180

Model 2: Multiple correlation coefficient (*R*) = 0.492; adjusted coefficient of determination (*R* ^2^) = 0.227

## Discussion

Masticatory movement is governed by the coordinated functions of oral organs: teeth, jaw, cheek, lips, and tongue. Among them, the tongue plays an important role in mastication and swallowing since it transports food to the molars, initiates mastication, mixes foods with saliva, and propels a food bolus into the pharynx. Furthermore, the swallowing reflex occurs because the tongue and the soft palate close at the region of the fauces. Many elderly people under long-term care develop malnutrition because of a decline in masticatory and swallowing functions as described above. Improvement in swallowing is considered the most effective way to treat dysphagia because oral dysfunction is also strongly associated with dysphagia [1]. Therefore, evaluating tongue dysfunction or abnormality may be an essential diagnostic procedure for dysphagia. There are many methods for evaluating tongue function, i.e., measuring the strength [14–17] and speed and location of movement [18]. The strength of the tongue has been evaluated by measuring the maximum tongue pressure against the palate [14, 15]. There are some reports that tongue function in the elderly declines with age [14, 15, 19, 20]. However, the effects of malnutrition on tongue volume in the elderly are still unknown. In our study we used ultrasonography to measure tongue thickness. Ultrasonography is widely used for functional analysis of dysphagia and is also reported to be very practical for anatomical analysis [21]. Furthermore, ultrasonography has enormous potential for visualizing the tongue in clinical research because it is noninvasive and it is easy to perform repeated examinations.

The age-associated loss of both muscle mass and strength, termed sarcopenia, is highly relevant to nursing home residents [22]. It was reported that tongue sarcopenia was observed more frequently in aged rats than in control rats [23, 24]. However, the relationship between tongue sarcopenia and aging in humans is obscure. The absence of occlusal support affects tongue movement and oral function [14, 25, 26]. In this study we employed subjects with posterior occlusal dentition of their natural teeth or dentures to eliminate confounding variables.

It has been suggested that TSF and AMA correlate with nutritional status [8, 10]. TSF represents fat volume and AMA the muscle volume of the upper arm. Since there was a significant association between tongue thickness and nutritional status, tongue muscle volume may also be related to nutritional status.

Furthermore, it was suggested that sarcopenia may develop not only in skeletal muscles but also in the tongue. Hence, dysphagia, tongue disuse syndrome, or malnutrition may affect tongue thickness, with subsequent worsening of malnutrition. Moreover, Saito et al. [27] reported that in rats, the structures of tongue muscles (genioglossus and geniohyoid) may be affected by fat deposition in myofibers. Determination of the fat fraction may be required in our future studies on tongue sarcopenia [28].

It was suspected that tongue thickness correlates with mandibular length. In this regard, an animal study [29] showed the relationship between tongue thickness and mandibular length from infancy through childhood, whereas no such relationship was identified in a human study [30]. However, in the present study we demonstrated a significant relationship between tongue thickness and AMA (an index indicating muscle mass) and age by applying multiple regression analysis. Neither HT, a marker of bone in humans, nor BW (a similar marker) was found to correlate with tongue thickness, suggesting that general muscle volume and/or age alone may affect this feature of the tongue.

Atrophy of the tongue may not be the only reason for reduced tongue function and inability to maintain nutritional status. However, Kikutani et al. [31] reported that oral functional training to maintain and/or improve feeding function is very efficient for improving the nutritional condition. It was reported that muscle is replaced by fat or fibrous tissues with aging [32], implying that tongue exercise might restore muscle tissue. Robbins et al. [32] and Yeates et al. [33] also reported that exercising the tongue prevented general sarcopenia. Therefore, effective measures or protocols to prevent malnutrition, which involve tongue exercise or rehabilitation, may be necessary to improve tongue disuse syndrome. For this purpose, our method of monitoring tongue thickness by ultrasonography may provide information for a tongue exercise protocol or treatment plan. We will study further the relationship between tongue pressure and tongue thickness in a future investigation.

## Conclusion

The findings of this study suggest that tongue thickness is related to nutritional status in the elderly.
